# The relationships between perceived individual and team characteristics, individual and team learning activities with effectiveness in nursing teams

**DOI:** 10.3389/fpsyg.2023.1163494

**Published:** 2023-04-26

**Authors:** Veronika Anselmann, Jasperina Brouwer, Regina H. Mulder

**Affiliations:** ^1^Institute of Nursing Science, University of Education Schwäbisch Gmünd, Schwäbisch Gmünd, Germany; ^2^Educational Sciences, Faculty of Behavioural and Social Sciences, University of Groningen, Groningen, Netherlands; ^3^Faculty of Human Sciences, University of Regensburg, Regensburg, Bavaria, Germany

**Keywords:** team learning, learning activities, knowledge sharing, psychological empowerment, nursing

## Abstract

**Introduction:**

Team learning plays a crucial role in addressing the shortage of nurses and ensuring that there are enough trained and capable nurses available during times of crisis. This study investigates the extent to which individual learning activities (1) contribute to knowledge sharing in teams and (2) impact the effectiveness of nursing teams. Furthermore, we want to obtain more insight into whether (3) the antecedents of individual psychological empowerment, teamwork preference, and team boundedness contribute to individual learning activities and knowledge sharing in nursing teams.

**Method:**

We conducted a cross-sectional questionnaire study of 149 gerontological nurses working in 30 teams in Germany. They completed a survey measuring knowledge sharing, teamwork preference, team boundedness, individual learning activities, psychological empowerment, and team effectiveness (as an indicator of performance).

**Results:**

The results from structural equation modeling revealed that individual learning activities contribute to knowledge sharing in teams and, as a result, enhance team effectiveness. In particular, psychological empowerment was found to be associated with individual learning activities, while teamwork preference and team boundedness were related to knowledge sharing.

**Discussion:**

The results indicated that the accomplishment of individual learning activities plays an important role in nursing teams, as it is linked to knowledge sharing and, as a result, contributes to team effectiveness.

## 1. Introduction

The current shortage of nurses worldwide (World Health Organization [WHO], 2020) highlights the importance of individual learning activities and their impact on team effectiveness. In light of this shortage, it is imperative for experienced nurses to educate their novice colleagues rapidly. Nurses are often assigned to various work environments and are required to collaborate with teams that are not their usual colleagues. Nurses effectively navigate such situations by incorporating diverse learning opportunities and gaining hands-on experience from their other colleagues. Both of these approaches may contribute to team effectiveness (VanDevanter et al., 2014). King et al. (2022) claimed that many nurses deployed during the COVID-19 pandemic reported that they could meet standards; however, some expressed concerns about the quality of health care. The availability of diverse resources for knowledge sharing and support can help individuals manage their responsibilities and overcome the challenges posed by various deployments (VanDevanter et al., 2014).

Nursing means working in teams (Anselmann and Disque, 2022). A nursing team is defined as “two or more nursing staff who work together to provide care and administrative tasks for a group of patients” (Kalisch et al., 2009, p. 3803). Team boundedness and teamwork preference reflect an individual’s sense of belonging to a team and their willingness to work in a team. Broetje et al. (2020) found that working in teams and having interpersonal relationships are important resources for nurses to handle the demands of their jobs. Therefore, for nurses, having a supportive and trustworthy team is a key factor in fostering effective teamwork (McInnes et al., 2015).

Teamwork is an important facilitator of performance in nursing teams (Schmutz et al., 2019). Defining nursing performance is challenging because nursing is a complex area with several co-occurring factors that impact performance (McCance et al., 2012). Nevertheless, nursing performance can be measured in terms of organizational factors involved in nursing healthcare provision (Dubois et al., 2013). Efficiency is one of the most important performance indicators because it “requires the formation of therapeutic relationships between professionals, patients, and others significant to them in their lives.” (McCance et al., 2012, p. 1149). Therefore, in line with Wageman et al. (2005), we focused on the concept of effectiveness as an indicator of performance in terms of nursing teams’ attainment of goals and attainments with regard to cost and time.

Although Tanyaovalaksna and Li (2013) observed that individual learning, team learning, and organizational learning are strongly interconnected, many studies have focused solely on team learning and team activities and have not specified the impact of individual learning activities on team learning and effectiveness (e.g., Timmermans et al., 2012). Rashkovits and Drach-Zahavy (2017), for example, showed that team accountability is positively associated with team learning and, hence, team effectiveness. Previous research, however, did not fully overlook the role of an individual in team learning and showed that individual characteristics (i.e., gender, education, and empowerment) and positive beliefs about teamwork preference, team learning, and improvement are important for team learning (Timmermans et al., 2012). Little is known about how accomplishments are derived from individuals’ learning activities within teams and how individual and team-related factors influence team learning activities (Timmermans et al., 2012).

**FIGURE 1:**
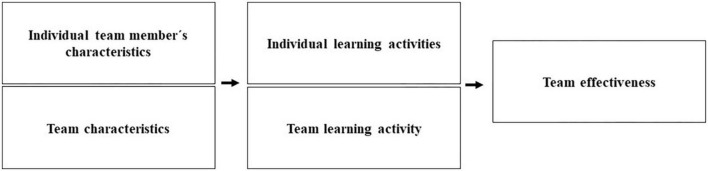
Research model.

Furthermore, research has shown that different conditions can influence team learning in various ways (Wiese and Burke, 2019). Therefore, in addition to individual learning activities and knowledge sharing, we also included the antecedents of psychological empowerment, teamwork preference, and team boundedness while investigating how knowledge sharing contributes to team effectiveness (see Figure 1). To sum up, there is a dearth of information on how individual learning is related to team learning activities and how these learning activities can be influenced.

Therefore, the following research questions were answered in this study:

(1) To what extent is psychological empowerment related to nurses’ individual learning activities?
(2) To what extent are team boundedness and teamwork preferences related to the team learning activity of knowledge sharing?
(3) To what extent does knowledge sharing relate to effectiveness?

We hypothesized that individual and team learning characteristics are related to individual and team learning activities as well as team effectiveness. We were interested in nurses’ perceptions of their individual and team conditions for their engagement in individual and team learning activities and their self-reported effectiveness.

Nurses are the largest group of professionals in healthcare systems worldwide (Labrague et al., 2022). Nurses are of great importance for obtaining more insights into how teamwork can be promoted in healthcare. Nurses work in teams to handle their complex work tasks.

In the second section, the theoretical framework will describe definitions of learning and individual and team learning activities. All components of the empirical study, such as the sample and instrument, are described in methodology in the third section, followed by an overview of the results in the final sections.

## 2. Theoretical framework

### 2.1. Informal learning

Simons and Ruijters (2004, p. 210) described learning as “implicit or explicit mental/or overt activities and processes leading to changes in knowledge, skills, or attitudes or the ability to learn from individuals, groups, or organizations.” Eraut (2004) described a continuum of informal and formal learning and defined informal learning as implicit, unintended, and unstructured learning. Because these learning processes are unstructured and experiential, they are often influenced by the learner’s intentions and preferences (Marsick and Volpe, 1999). Vygotsky’s (1978) sociocultural theory defines learning as a socially mediated process. Social interactions with team members can lead to cognitive development. The context in which social practices can be embedded is important for learning (Vygotsky, 1978).

Informal learning “includes the relations and dynamics among individual learners and learning collectives and is often embedded in everyday practice” (Lundgren et al., 2017, p. 317). This idea is in accordance with Dechant et al.’s (1993) definition of learning as an interaction between individuals, team beliefs, values, norms, and knowledge sharing. Informal learning activities can be socially shared or performed individually, independent of the context in which they are accomplished. Individuals can have ideas and gain knowledge and information for their own edification or share them with others. Informal learning activities occur in individual cognitive processes but can be shared and, through this sharing, become social learning activities (Russ-Eft et al., 2014). Participating in team learning activities is one way that nurses can handle the demands of their job.

Individual and team learning activities can be influenced by factors at the individual and team levels. Research has shown that psychological empowerment, as someone’s estimation of the importance of work (Seibert et al., 2011), is an “important internal incentive factor” for nurses’ motivation (Li et al., 2018, p. 1265).

### 2.2. Team learning

Decuyper et al. (2010, p. 116) developed a “systemic, cyclical and integrative team learning model that organizes and combines team learning processes, outputs, inputs, catalyst emergent states, and time-related variables into a coherent whole.” Team learning is a dynamic process (Edmondson, 1999; Decuyper et al., 2010) in which team members engage in group learning activities. Knowledge sharing is a fundamental team learning activity and can be described as “communicating knowledge, competencies, opinions, or creative thoughts from one team member to the other team member” (Decuyper et al., 2010, p. 116). Sharing knowledge in teams is required to develop strategies and determine productive and innovative solutions for work tasks (Timmermans et al., 2012).

### 2.3. Individual learning activities in the team context

The “multi-level” model by Decuyper et al. (2010, p. 117) shows that team learning activities, such as knowledge sharing, can be influenced by team members’ behaviors and characteristics. Individuals engage in informal learning activities, particularly when they encounter critical situations that require problem-solving or finding solutions to work-related problems (Marsick and Watkins, 2001; Manuti et al., 2015). As such, informal learning activities are not necessarily planned (Eraut, 2004) but can arise as a “by-product of work activities” (Joeng et al., 2018, p. 130) that are “self-directed, intentional, and field-based” (Cerasoli et al., 2018, p. 2). They can be differentiated from individual and social learning activities (Mulder, 2013). Individual learning activities are carried out by an individual and may result in individual learning outcomes, which might then be shared as knowledge among team members. Social learning activities, such as knowledge sharing, are carried out in interaction with others and lead to output within a social setting. Learning activities can be mental and covert or physical and overt (Simons and Ruijters, 2004). All of these characteristics need to be considered when investigating learning activities.

### 2.4. Conditions for nurses’ engagement in individual and team learning activities

The characteristics of team members can serve as preconditions for learning activities (Decuyper et al., 2010, p. 122). One individual characteristic that is particularly important is psychological empowerment (Jha, 2019). Psychological empowerment is defined as someone’s estimation of the importance of work and his or her motivation to take the initiative and handle work situations (Seibert et al., 2011). According to Spreitzer’s (1995) multidimensional instrument, psychological empowerment consists of four components: meaning, self-determination, belief in one’s own competencies, and belief in impact. Meaning refers to one’s beliefs and values and how they align with the work environment. Self-determination refers to having control over one’s work tasks and actions in the workplace. Competence concerns one’s belief in their ability to successfully handle work tasks. The fourth component, belief in impact, is one’s conviction that they have an influence on work actions and performance (Kraimer et al., 1999; Seibert et al., 2011). A condition that is especially relevant to work teams is team members’ preference for working in teams. Teamwork preference is considered an individual’s orientation toward a group and an individual’s attitude toward the work within the context of their team (Kiffin-Petersen and Cordery, 2003).

In addition, emergent states of a team—such as one’s feeling of belonging to the team—are an influential factor in team learning activities (Decuyper et al., 2010). Team boundedness contributes to team cohesion, which can enhance members’ willingness to share knowledge since they will not consider it “too costly” (Dey and Ganesh, 2020). Conversely, when the boundaries of a team are blurred, trust and cohesion may decrease (Mortensen and Haas, 2018), leading to less willingness to share knowledge.

### 2.5. Team effectiveness

These conditions and consequential team learning activities determine a team’s performance (Decuyper et al., 2010). Assessment of nursing performance is often based on a patient’s estimation of their received quality of care and the achievement of organizational goals (Germain and Cummings, 2010). In contemporary society, the healthcare system is strongly affected by technical and organizational changes, financial difficulties (Germain and Cummings, 2010), and a lack of qualified nursing staff (DeLucia-Waack, 1997). The assessment of nursing performance can be based on the measurement of a nurse’s competencies, patient outcomes, or the nurse’s behavior or competencies in specific situations or with regard to specific tasks such as triage decisions (DeLucia-Waack, 1997). In the present study, the focus was on the effectiveness of nursing teams. Wageman et al. (2005) defined the effectiveness of a team as the attainment of goals and expectations with regard to cost and time. Team effectiveness describes the productive outcome of a team and the output that meets its intended purpose (Hoegl and Gemuenden, 2001; Wageman et al., 2005). Sharing information with team members can contribute to team effectiveness (Anselmann and Mulder, 2020).

Figure 1 visualizes the assumed relationships between individuals and team characteristics as conditions for individual and team learning activities and their relationship to team effectiveness.

The following hypotheses will be tested:

H1: Psychological empowerment positively relates to individuals’ engagement in individual learning activities.

H2: Teamwork is positively preference related to knowledge sharing.

H3: Team boundedness is positively related to knowledge sharing.

H4: Engagement in individual learning activities is positively related to knowledge sharing.

H5: Knowledge sharing is positively related to team effectiveness.

## 3. Materials and methods

### 3.1. Sample and procedure

We conducted a cross-sectional questionnaire study. The participants in our study included 149 gerontological nurses (91% female) with a mean age of 42 years (*M* = 42.07; SD = 12.97). On average, the respondents had more than 4 years of experience as gerontological nurses (*M* = 4.88; SD = 1.29 years). They worked in 30 different gerontological nursing teams (*N* = 30, *n* = 149) at 17 retirement homes in Bavaria, Germany. The sizes of the retirement communities varied from small (more than 65 clients) to large (more than 100 clients). The size of the nursing teams varied from 3 to 12 nurses. The survey data were collected with paper and pencil, and the survey items were written in German. Participants were informed about the aims of the research project and its measurements, and their participation was voluntary. We started this research project in 2016. With respect to the work of nurses, the German healthcare system has many similarities with other healthcare systems in other European countries. For instance, nurses in hospitals and retirement homes have to work together in teams, which makes this study valuable for other countries.

### 3.2. Measures

In addition to background variables (i.e., gender, age, years of experience, etc.), the present study used validated scales to measure the other variables. All variables showed satisfactory Cronbach’s alpha, ranging from 0.74 to 0.88, indicating good internal consistency (see Table 1).

**TABLE 1:** Mean, standard deviation, Cronbach’s alpha, and bivariate correlations.

Scales	M	SD	α	1	2	3	4	5
1. Individual learning activities				–				
2. Knowledge sharing	3.97	0.65	0.88	0.42**	–			
3. Psychological empowerment	4.15	0.65	0.86	0.46**	0.14	–		
4. Teamwork preference	4.16	0.77	0.87	0.29**	0.16	0.27*	–	
5. Team boundedness	4.08	0.90	0.74	0.29**	0.13	0.27**	0.36**	–
6. Effectiveness	3.86	0.62	0.84	0.26**	0.33**	0.27**	0.26**	0.33**

*p < 0.05, **p < 0.001.

Spreitzer (1995) developed a 12-item multidimensional assessment of psychological empowerment in the work environment, which was used in this study. Four subscales measure meaning, competence, impact, and self-determination. Example items include the following: “My work is really important for me,” “I am confident that I have the skills to perform my job,” “I can determine to a large extent how I can perform my job,” and “I can control what happens in my job.” The answering format was a 5-point Likert scale ranging from 1 = “absolute agreement” to 5 = “disagreement.”

Individual learning activities were measured with a list of 24 learning activities. Out of 24 learning activities, 12 were individual and 12 were social. This approach for measuring individual learning activities was developed by Mulder (2013). The participants were asked to estimate how often they fulfilled the listed learning activities with an answer format in the form of a 5-point Likert scale that ranged from 1 = “never” to 5 = “very often.” The list of learning activities is a list of possible activities, indicating that individuals engage in different learning activities. We counted the number of learning activities participants were assigned to indicate their engagement in learning activities. For instance, an individual mental learning activity is “thinking about specialized literature,” whereas an individual physical learning activity is “searching on the Internet.” An example of a mental, social learning activity is “thinking together with a colleague about the support received.” For instance, physical and social activity is “getting information from a person outside of the team” (see Messmann and Mulder, 2015).

Knowledge sharing was measured using Staples and Webster’s (2008) instrument, with a 5-point Likert scale answering format ranging from 1 = “absolute agreement” to 5 = “no agreement.” This measures a nurse’s perception of their knowledge of a colleague. Example items are “People in this team are willing to share knowledge/ideas with others” and “People in this team share their ideas openly” (Staples and Webster, 2008, p. 639).

Teamwork preference referred to an individual’s attitude toward working together with others in teams (Kiffin-Petersen and Cordery, 2003) and was measured with three items and a 5-point Likert scale ranging from 1 = “absolutely” to 5 = “not at all.” An example item is, “I appreciate working in a team.”

Team boundedness was measured with Wageman et al.’s (2005) three items. Nurses responded in the answer format of a 5-point Likert scale ranging from 1 = “absolutely” to 5 = “not at all.” One example item is: “The team is stable; there is no cast change.”

Team effectiveness, which is an individual’s perception of the extent to which a team achieves its objectives, was measured with five items designed by Van Woerkom and Croon (2009), with a 7-point Likert scale answer format ranging from 1 = “strongly agree” to 7 = “strongly disagree.” An example item is: “As a team, we achieve our goals.” In this study, we were interested in the individual team members’ perceptions of their teams’ effectiveness, which required self-reports. Following Ajzen (1991), we were interested in nurses’ estimation of their team’s performance because it can be assumed that these perceptions can influence behavior.

### 3.3. Data analyses

Descriptive statistics and correlation analyses were conducted using SPSS 25.0. Furthermore, we used structural equation modeling to test the assumed hypotheses. We used Mplus 6 (Muthén and Muthén, 2012). Different characteristics needed to be considered in the analyses. First, knowledge sharing was estimated using the individual participants’ views of knowledge sharing in their teams. This enabled knowledge sharing to describe team learning activities. Second, because the participants were members of teams that were included in the data collection, the data were nested.

To meet these characteristics, we used an approach capable of considering complex data (cf. Marcoulides and Schumacker, 2009). By using this approach [i.e., Maximum Likelihood Robust (MLR) estimation and type complex], data at the individual level could be analyzed, and the clustering and nestedness of the data were considered. The study used fit indices described by Kline (2010). An acceptable fit was indicated by SRMR ≤ 0.10, CFI ≥ 0.90, and RMSEA ≤ 0.08. For psychological empowerment and individual learning activities, we had factors measuring different components of the variables so that we estimated both as second-order variables (Wickrama et al., 2021).

## 4. Results

In Table 1, the bivariate correlations among individual learning activities and knowledge sharing, teamwork preference, team boundedness, psychological empowerment, and team effectiveness are listed. Individual learning activities were significantly related to knowledge sharing (*r* = 0.42, *p* < 0.001). Psychological empowerment and individual learning activities were also significantly related (*r* = 0.46, *p* < 0.001). A relationship exists between team boundedness and teamwork preference (*r* = 0.27, *p* < 0.001). Effectiveness moderately correlates with knowledge sharing (*r* = 0.33, *p* < 0.001). Based on these results, we specified the structural equation model.

**FIGURE 2:**
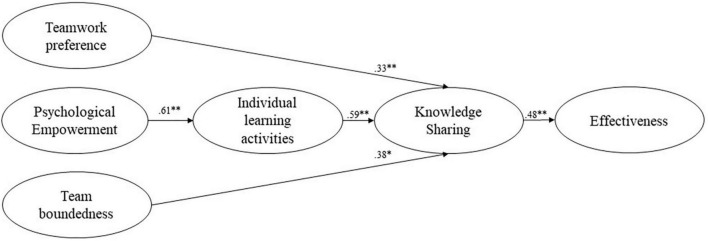
Structural equation model of psychological empowerment, individual learning activities, knowledge sharing, team boundedness, team work preference, and team effectiveness. *p < 0.05, **p < 0.001.

The structural equation model (Figure 2) shows an acceptable fit to the data (SRMR = 0.08, CFI = 0.92, RMSEA = 0.04). The results of the modeling indicate that psychological empowerment was related to individual learning activities (β = 0.61, *p* < 0.001). This result supports Hypothesis 1. We also found support for Hypotheses 2 and 3 since team boundedness (β = 0.38, *p* < 0.001) and teamwork preference (β = 0.33, *p* < 0.05) were related to knowledge sharing. The accomplishment of individual learning activities was positively related to the team learning activity of knowledge sharing (β = 0.48, *p* < 0.001), which supports Hypothesis 4. In addition, Hypothesis 5 was supported by the finding that knowledge sharing is positively related to team effectiveness (β = 0.59, *p* < 0.001).

## 5. Discussion

The present study contributes to the literature on team learning in the nursing field by testing a research model in which different conditions play an important role in team performance. The results highlight the importance of individual and team conditions for informal learning at the workplace. Although knowledge sharing has been investigated in organizations (Noe et al., 2014), the understanding of the role of individual learning activities and knowledge sharing in nursing teams is rather limited. Our results indicate that engagement in individual learning activities plays an important role in nursing teams because it links to knowledge sharing and, hence, to team effectiveness.

The results of this study show that knowledge sharing in teams is an important activity that is related to team effectiveness. This relation could be found in different other studies. Ahmad and Karim (2019) showed in their systematic review that knowledge sharing can influence outcomes such as performance at the individual, team, and organizational levels. The accomplishment of an individual learning activity is not directly related to the effectiveness of nursing teams. When knowledge is shared within the nursing team, it relates to the perceived team’s effectiveness. Our results are in line with a meta-analysis from Wiese et al. (2022). They found that “intrateam learning behaviors uniquely predict performance” (Wiese et al., 2022; p. 571). Intrateam learning behaviors can be defined as “internal behavioral processes that teams engage in that build shared meaning from existing information.” (Wiese and Burke, 2019, p. 4).

Wiese et al. (2022) showed that conditions such as a team’s familiarity or task complexity are unrelated to a team’s performance. The results of this study show that individual characteristics, such as team characteristics, are related to learning activities but not directly to team performance. When nurses prefer working in teams and perceive a close connection with their colleagues, this contributes to knowledge sharing among nurses, allowing an organization to use the existing knowledge of the individual nurses. These findings are consistent with Cabera and Cabera’s (2002) proposition to increase group identity and commitment to enhancing knowledge sharing. Dey and Ganesh (2020) found that team boundedness can have an impact on dynamics in teams. While studies in other domains revealed that flexible belonging to teams can positively affect a team’s performance (Dibble and Gibson, 2018), nurses need a feeling of belonging to a team to share their knowledge (Longacre et al., 2019).

Psychological empowerment is an individual resource and an antecedent for engaging in individual cognitive and physical learning activities. This is in line with the results of a study from Jha (2019), who found that psychological empowerment is related to a learning orientation. This can have an effect on team performance. Studies in nursing showed that nurses who felt high psychological empowerment had a lower intention to leave their job (Shapira-Lishchinsky and Benoliel, 2019).

### 5.1. Implications of this research

The current study’s findings provide insights into how individual and team learning activities are related and what conditions can have an influence on them. In this study, we were interested in team effectiveness as estimated by nurses. Their individual perception of meaning, competence, self-determination, and impact is not perceived as a condition for their team’s performance. It leads to their engagement in individual learning activities, which leads to knowledge sharing in teams. By doing so, they perceive that their teams’ performance can be increased.

While our study results show that individual characteristics are related to individual learning and team characteristics to team learning, we agree with Wiese et al. (2022) that there is a need for subsequent theory development and research.

The current study’s findings provide insights into how individual and team learning activities are related and the conditions involved. While our study results indicate that individual characteristics are related to individual learning and team conditions to team learning, we agree with Wiese et al. (2022) that there is a need for subsequent theory development and research on the relationship between conditions and learning activities.

Regarding psychological empowerment, Friend and Sieloff (2018) proposed a theory for nursing in which group empowerment is included. Our study results showed that empowerment can affect individual learning activities. Empowerment is considered an individual’s positive perception of having control over one’s work. This could increase collaboration and, by extension, team effectiveness. Contributing to this line of reasoning and based on our results, empowerment (in particular confidence in meaning, competence, self-determination, and impact) is an important resource and antecedent, and individual learning activities and knowledge sharing are crucial for team effectiveness in addition to team preferences and perceptions of team boundedness. Nurses seem to engage in individual learning activities when they feel capable of making work-related choices on their own that impact their work. Further research focuses on the team’s perception of empowerment and finds out how relations between team members can strengthen their performance.

Importantly, in addition to empowerment, work structures can impact nurses’ engagement in individual learning activities (Kalisch et al., 2009). Further research is required to increase insight into what kinds of work structures enhance individual learning activities.

Furthermore, research should work to gain further insight into the quality of shared knowledge and find out how team members’ knowledge is shared, for instance, in a network (cf. Brouwer and Jansen, 2019; Brouwer and Froehlich, 2020). Social network research can help investigate and inform us about with whom nurses exchange their knowledge or accomplish their social learning activities among their colleagues. It may prove interesting to gain more information about the types of knowledge that team members share by capturing them using a team mental model.

### 5.2. Implications for practice

The results are informative for nursing team leaders and managers, as they indicate that fostering individual learning activities and team learning activities are related to a team’s effectiveness. Results of our study showed that psychological empowerment, team boundedness, and teamwork preference are related to individual and to team learning activities. To improve team performance, team leaders and managers in nursing need to foster the engagement of individual and team learning activities by providing opportunities for these activities (through, for instance, fitting work structures and time for reflection, as well as fostering psychological empowerment and team boundedness).

Nurses need time to accomplish learning activities, meet with each other, and discuss relevant issues. For this reason, it is crucial to empower nurses, consider their preferences for working in a team, and enhance team boundedness. Team leaders should be aware of the importance of these factors, take these aspects into account, and foster them. This might require rethinking management styles (Nevalainen et al., 2018). In addition to creating possibilities for knowledge sharing, team leaders should pay attention to team members’ work preferences, psychological empowerment, and the team’s overall boundedness because these foster knowledge sharing in nursing teams.

### 5.3. Limitations

One limitation concerns the relatively small sample size within a cross-sectional design. Nevertheless, the model fit was acceptable. We recommend future studies investigate team learning with longitudinal designs to make temporal inferences and to obtain a better understanding of the changes over time in the accomplishment of the learning activities, knowledge sharing, and the effect on team effectiveness.

The study was performed on 30 teams in one specific sector within healthcare (gerontological nursing). Therefore, the study should be repeated in other healthcare sectors (for example, acute care) to improve the generalizability of the results.

In addition, the study focused on one performance indicator to measure nursing teams’ performance (i.e., team effectiveness). Other forms of team performance indicators could be used in further research, such as absenteeism, wellbeing, patient safety indicators, and reports of incidents (see Devasahay et al., 2021).

Finally, the data were collected through self-reports. With other instruments, such as interviews, focus groups (Merriam and Tisdell, 2016), or observations, more insight can be gained into (1) what exactly happens, (2) how and what kind of knowledge has been shared among team members, and (3) the meaning of the relationships between individual and team characteristics with learning activities, sharing knowledge, and team effectiveness; this might require a mixed-methods design. Our results showed that learning activities are related and can be assumed to be understood as a learning process. Therefore, more insights into learning as a process should be gained instead of investigating single activities. This could be realized using process approaches, such as time-series techniques (Poole and Van de Ven, 2004).

## 6. Conclusion

In nursing, learning is critical for coping with challenging, unexpected, and new situations. Informal learning involves both individual learning activities and knowledge sharing among nursing team members. Nurses’ individual empowerment is positively related to the accomplishment of individual learning activities. Engagement in individual learning activities does not seem to foster team effectiveness directly. More important for team effectiveness, it seems, is that the team members share their knowledge.

## Data availability statement

The datasets presented in this article are not readily available because data could not be made applicable because this was not part of participants written informed consent. Requests to access the datasets should be directed to VA, veronika.anselmann@ph-gmuend.de.

## Ethics statement

Ethical review and approval was not required for the study on human participants in accordance with the local legislation and institutional requirements. The patients/participants provided their written informed consent to participate in this study.

## Author contributions

VA and RM contributed to the conception and design of the study. VA collected the data and performed the statistical analysis. VA and JB wrote the first draft of the manuscript. RM wrote sections of the manuscript and gave feedback. All authors contributed to manuscript revision, read, and approved the submitted version.
